# Machine Learning and Deep Learning Methods for Fast and Accurate Assessment of Transthoracic Echocardiogram Image Quality

**DOI:** 10.3390/life14060761

**Published:** 2024-06-13

**Authors:** Wojciech Nazar, Krzysztof Nazar, Ludmiła Daniłowicz-Szymanowicz

**Affiliations:** 1 Faculty of Medicine, Medical University of Gdansk, Marii Sklodowskiej-Curie 3a, 80-210 Gdansk, Poland; 2 Faculty of Electronics, Telecommunications and Informatics, Gdansk University of Technology, Gabriela Narutowicza 11/12, 80-233 Gdansk, Poland; s184698@student.pg.edu.pl; 3 Department of Cardiology and Electrotherapy, Faculty of Medicine, Medical University of Gdansk, Smoluchowskiego 17, 80-213 Gdansk, Poland; ludmila.danilowicz-szymanowicz@gumed.edu.pl

**Keywords:** echocardiography, machine learning, artificial intelligence, image quality, classification, convolutional neural networks

## Abstract

High-quality echocardiogram images are the cornerstone of accurate and reliable measurements of the heart. Therefore, this study aimed to develop, validate and compare machine learning and deep learning algorithms for accurate and automated assessment of transthoracic echocardiogram image quality. In total, 4090 single-frame two-dimensional transthoracic echocardiogram images were used from apical 4-chamber, apical 2-chamber and parasternal long-axis views sampled from 3530 adult patients. The data were extracted from CAMUS and Unity Imaging open-source datasets. For every raw image, additional grayscale block histograms were developed. For block histogram datasets, six classic machine learning algorithms were tested. Moreover, convolutional neural networks based on the pre-trained EfficientNetB4 architecture were developed for raw image datasets. Classic machine learning algorithms predicted image quality with 0.74 to 0.92 accuracy (AUC 0.81 to 0.96), whereas convolutional neural networks achieved between 0.74 and 0.89 prediction accuracy (AUC 0.79 to 0.95). Both approaches are accurate methods of echocardiogram image quality assessment. Moreover, this study is a proof of concept of a novel method of training classic machine learning algorithms on block histograms calculated from raw images. Automated echocardiogram image quality assessment methods may provide additional relevant information to the echocardiographer in daily clinical practice and improve reliability in clinical decision making.

## 1. Introduction

Echocardiography is an essential tool in clinical diagnosis, accurate treatment, and patient prognosis prediction; therefore, high-quality echocardiogram images are the cornerstone of accurate and reliable measurements of heart structures [1,2,3,4]. In the field of echocardiography, advanced AI-based computerized measurement methods are being intensively developed [1,2,3,5,6]. Technologies such as semi-automated left ventricle ejection fraction (LVEF) or global longitudinal strain (GLS) calculations are now widely used in daily clinical practice to guide clinical decisions regarding patient appropriateness for medical and interventional therapies as well as routine monitoring of the resulting clinical outcomes [7,8,9]. They are also used to perform follow-ups in cohort studies and randomized controlled trials [8,9,10]. The constantly growing dependency of echocardiography on sophisticated mathematical models may lead to the discovery of new diagnostic and prognostic methods and help develop precise, personalized and holistic medicine [5,11,12]. Moreover, high automation of measurements may aid in reducing human error and the number of measurement steps [5,7,11,12]. This will improve the objectiveness of the analysis as well as save time in echocardiographic workflow. Overall, using AI-enabled echocardiography may bring rapid improvement in the quality of patient care and medical research and result in a colossal change for the better [12].

However, the reliability of advanced machine learning and human-derived echocardiographic measurements will always depend on the echocardiogram image quality [3]. The lower the image quality, the greater the inter-observer variability in such important parameters as the LVEF and GLS [1]. Moreover, automated test–retest GLS measurements using speckle-tracking artificial intelligence (AI) algorithms depend on image quality, too [2,3]. In addition, echocardiograms with poor image quality show a lower correlation between automated echocardiographic analysis and reference measurements calculated using cardiac magnetic resonance [2]. Poor-quality images may also lead to false clinical diagnoses of cardiac dysfunction in some clinical situations [2].

In comparison to many studies that aim to highlight the potential benefits of the use of AI and the development of AI models [5,11,12,13,14,15,16], only Sengupta et al., Huang et al., Nagata et al., and Saikhan et al. analyzed the impact of image quality on the accuracy of clinically relevant echocardiographic measurements and outcomes regarding diagnosis and prognosis [1,2,3,4]. Moreover, a small number of studies aim to predict image quality from two-dimensional raw, unprocessed echocardiogram images [17,18,19]. All of them use convolutional neural networks [17,18,19].

Convolutional neural networks are one of the most sophisticated mathematical modelling tools that significantly improve prediction accuracy in various fields [20,21,22]. However, compared to less advanced traditional machine learning algorithms, other than neural networks, their training and validation require more computational power and time [14]. Thus, exploring additional machine learning tools, methods and techniques within this research area is valuable. Classic machine learning usually uses tabular data to predict a given end-point [23,24]. A tabular data set for an image could be its histogram, defined as the frequency of occurrence of each brightness value from 0 to 255 [25]. Next, a classic machine learning algorithm could use the histogram data to predict the image quality.

Overall, automated evaluation of the echocardiogram image will lead to a more objective estimation of the image quality indices [19]. Such a system can aid in the training of new echocardiographers. Moreover, automated analysis of large image datasets could support medical researchers in the inclusion/exclusion of individual patients in future clinical studies. Finally, this tool may provide additional relevant information to the echocardiographer in daily clinical practice and may improve reliability in clinical decision making.

### Aim

To develop, validate, and compare machine learning and deep learning algorithms for accurate, automated, and objective transthoracic echocardiogram image quality assessment.

## 2. Materials and Methods

### 2.1. Materials

In total, 4090 single-frame two-dimensional transthoracic echocardiogram images from apical 4-chamber, apical 2-chamber and parasternal long-axis views were used. The data were extracted from two free open-source datasets [15,26].

### 2.2. CAMUS Dataset

**Figure 1:**
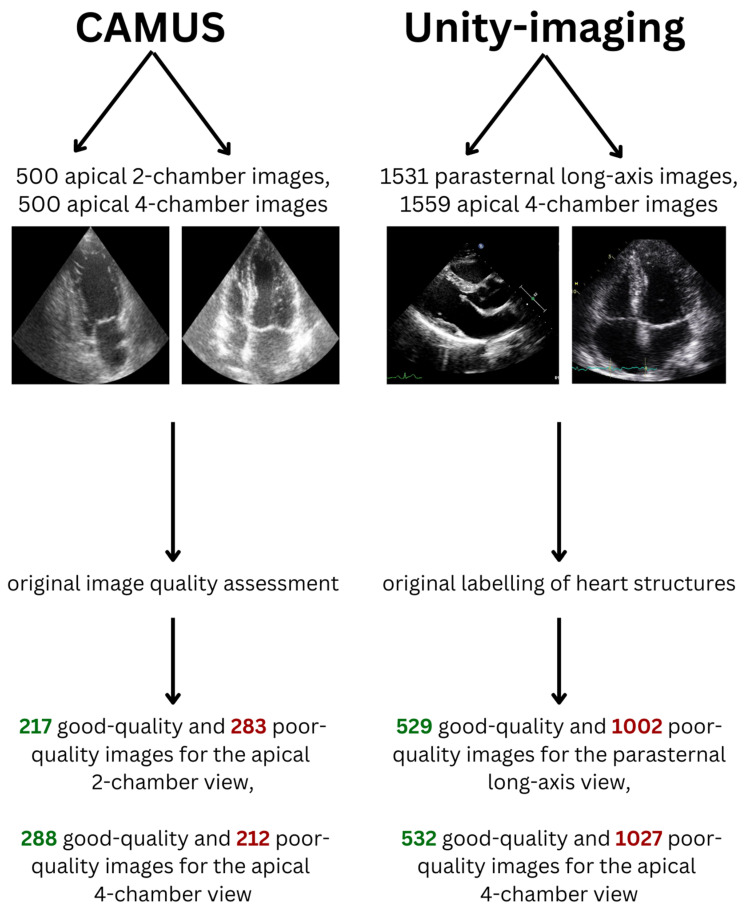
Datasets included in the study.

Five hundred apical 4-chamber and 500 apical 2-chamber end-diastolic images from 500 patients were extracted from the CAMUS dataset (Figure 1, Table 1). This dataset was originally provided to resolve the problem of echocardiographic image segmentation and volume estimation [26]. For every image in this dataset, its quality was assessed by an experienced cardiologist and was labelled as “good”, “medium” or “poor”.

For this study, to make the classes in the dataset more balanced and to perform binary image quality classification, medium- and poor-quality images were considered as poor-quality images. In total, there were 217 good-quality and 283 poor-quality images for the apical 2-chamber view dataset (43.4% and 56.6% of the dataset, respectively). Moreover, there were 288 good-quality and 212 poor-quality images for the apical 4-chamber view dataset (57.6% and 42.4% of the dataset, respectively).

### 2.3. Unity Imaging Collaborative Dataset

This dataset contains 7523 echocardiographic images in parasternal long-axis and apical views and was created for the development and validation of AI in cardiology [15]. Every image in this dataset contains a set of labels of clinically relevant structures that can be located and used for the measurement of cardiac chambers. For every image, each label was described in the original dataset as “off” (if the structure was not fully present in the image), “blurred” (if the structure was too blurry to provide an exact location in the image, but was fully present) or “point”/“curve” (if it was possible to locate the structure accurately—it was fully present in the image and the borders depicting the structure were not blurred). The structures were originally labelled by at least one expert in the field.

For the purpose of this study, labels described as “off” received 0 points, “blurred” received 1 point and “point”/“curve” received 3 points. When there were many similar images for one patient for a given view only one randomly chosen image was included in the final datasets used in our study. In total, 1531 parasternal long-axis and 1559 apical 4-chamber images from 3030 patients were extracted from this dataset. The Unity Imaging dataset did not specify in the original data whether the frames are end-diastolic or end-systolic. For the apical 4-chamber view 21 structures were considered relevant and for the parasternal long-axis view 44 structures were analyzed. In a given echocardiographic view, a structure was considered “relevant” if it could be technically visualized in that projection. Next, for every image dataset, a median number of points received by the images in this dataset was calculated. Images that received fewer points than the median or median number of points were considered poor-quality (since a limited number of structures could be identified and measured), while images that had higher than the median number of points were considered good-quality (Table 1). Therefore, in total, there were 529 good-quality and 1002 poor-quality images for the parasternal long-axis view dataset (34.6% and 65.4% of the dataset, respectively). The median cut-off value was 55. Moreover, there were 532 good-quality and 1027 poor-quality images for the apical 4-chamber view dataset (34.1% and 65.9% of the dataset, respectively). The median cut-off value was 36. The skewed distribution resulted from the fact that many images received exactly the median number of points.

There were fewer than 300 single-patient apical 2-chamber and apical 3-chamber images and, therefore, they were not included in the development of AI algorithms.

### 2.4. Methods

This study was conducted following the framework of the Proposed Requirements for Cardiovascular Imaging-Related Machine Learning Evaluation (PRIME) [27].

### 2.5. Histogram Dataset

For every raw echocardiogram image, additional grayscale block histograms were developed. For this purpose, every raw image in its native resolution was divided into blocks. The blocks were geometrically represented as a square grid (square tilting). The number of blocks per image was equal to a square of grid length. The grid lengths examined in the study were equal to 1, 3, 5, 8 and 10. Thus, the number of blocks per image ranged from one (for a grid length equal to 1) to a hundred (for a grid length equal to 10). A histogram, defined as the frequency of occurrence of each brightness value from 0 to 255 [25], was developed for every block. Next, to reduce computing time, every consecutive five values in the generated histogram were averaged. To make the block histograms comparable in between images, the absolute counts of values in the histogram were then divided by the sum of all values in the histogram. Thus, the sum of all values in every block histogram was equal to 1. For every grid length, a resulting dataset of block histograms was then used for the development of non-deep learning algorithms (classic machine learning).

### 2.6. Machine Learning

**Figure 2:**
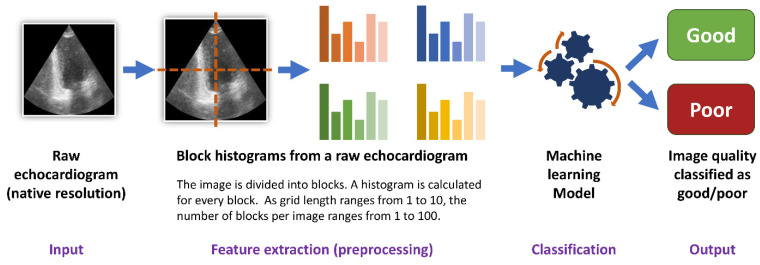
Training of the machine learning algorithms.

For every histogram dataset 6 classic machine learning algorithms were tested (Random Forest, AdaBoost, Support Vector classifier, Decision Tree, K-Neighbors and XGBoost). All algorithms were developed for all histogram grid lengths (from 1 to 10) and were tested with the use of stratified 5-fold cross-validation (Figure 2). Every model performed a binary classification of the data with the use of their default hyperparameters.

### 2.7. Deep Learning—Convolutional Neural Networks

**Figure 3:**
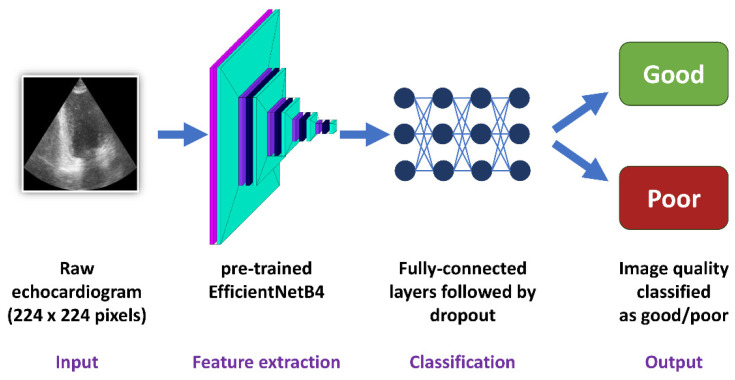
Training of the convolutional neural networks.

For every image dataset, a convolutional neural network with the use of the pre-trained EfficientNetB4 architecture was developed (Figure 3). This architecture was chosen because it has one of the best ratios of accuracy to training time and model complexity [22]. Moreover, the use of the pre-trained state-of-the-art network architecture not only improved the stability of the training but also allowed for high classification accuracy as well as reliability and credibility of the obtained results. The image input size was 224/224/3. The feature extraction block (convolutional block) consisting of the convolutional layers of EfficientNetB4 with ImageNet weights was followed by a flattening layer. Next, the classification block included four fully connected layers with 512 neurons, each followed by a Dropout of 0.25. The last layer contained two neurons responsible for the results’ binary classification. An Adam optimizer with a learning rate equal to 0.0001 was used. All models were evaluated with the use of stratified 5-fold cross-validation.

### 2.8. Statistical Analysis

Continuous data were summarized with the use of means and standard deviations. The developed algorithms’ accuracy, sensitivity, specificity, and area under the receiver operating curve (AUC) were summarized as percentages. Moreover, receiver operating curves (ROCs), true positive, true negative, false positive and false positive values from confusion matrixes were also calculated. The chi-square test with Yates’ correction was used to analyze the differences in accuracies of the presented AI models. The threshold of statistical significance was set at *p* < 0.05. Additionally, 95% lower and upper confidence intervals (95% CIs) were also computed.

All analyses were performed using Python 3.10.9 and Numpy, 1.23.5, Pandas 2.1.3, Scikit-learn 1.2.1, XGBoost 2.0.1, Keras 2.10 and Tensorflow 2.10 libraries.

## 3. Results

### 3.1. CAMUS Apical 2-Chamber View

**Figure 4:**
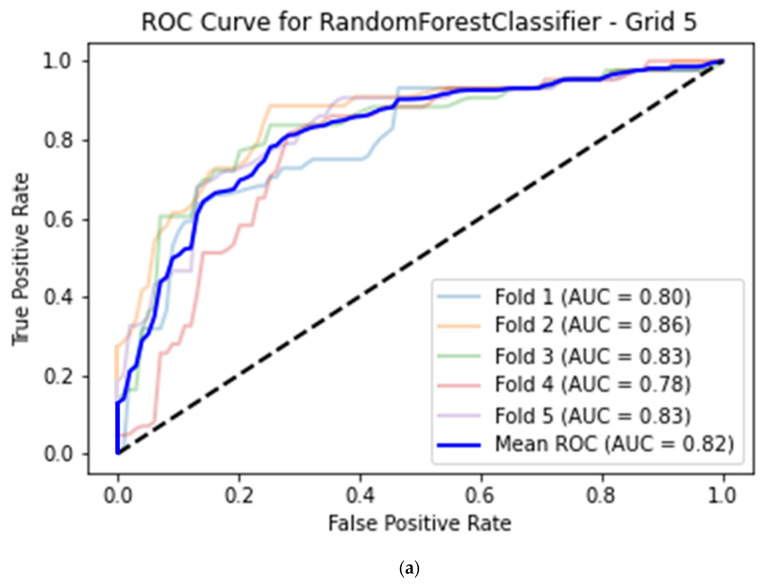

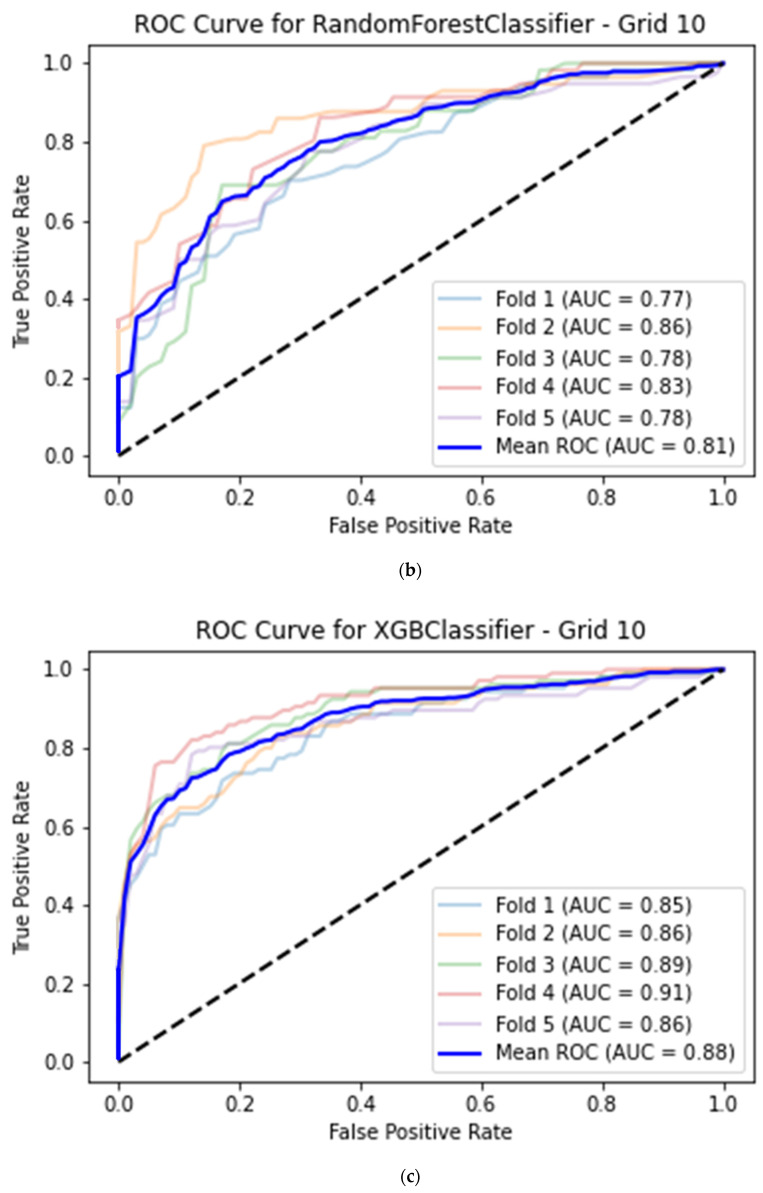

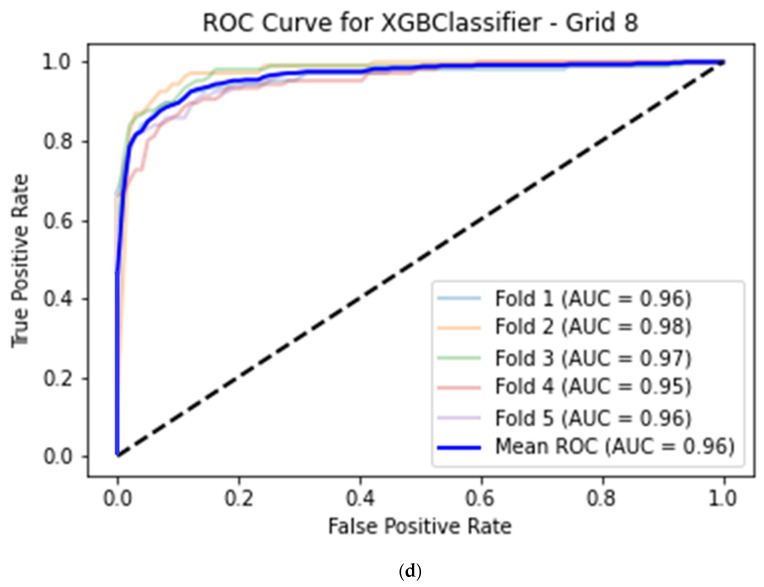
(**a**) Best model for CAMUS apical 2-chamber view image quality classification—Random Forest classifier (histogram grid length 5) with Receiver Operating Curve. AUC—area under the curve. (**b**) Best model for CAMUS apical 4-chamber view image quality classification—Random Forest classifier (histogram grid length 10) with Receiver Operating Curve. AUC—area under the curve. (**c**) Best model for Unity Imaging parasternal long-axis view image quality classification—XGBoost classifier (histogram grid length 10) with Receiver Operating Curve. AUC—area under the curve. (**d**) Best model for Unity Imaging apical 4-chamber view image quality classification—XGBoost classifier (histogram grid length 8) with Receiver Operating Curve. AUC—area under the curve.

For the best machine learning model, the average classification accuracy of image quality for the histogram dataset calculated for the apical 2-chamber view was equal to 0.76 (95% CI 0.72–0.79) with 0.68 (95% CI 0.64–0.72) sensitivity, 0.82 (95% CI 0.78–0.85) specificity and 0.82 (95% CI 0.78–0.85) AUC. This model was based on a Random Forest classifier with a histogram grid length of 5 (Table 2, Figure 4a).

**Figure 5:**
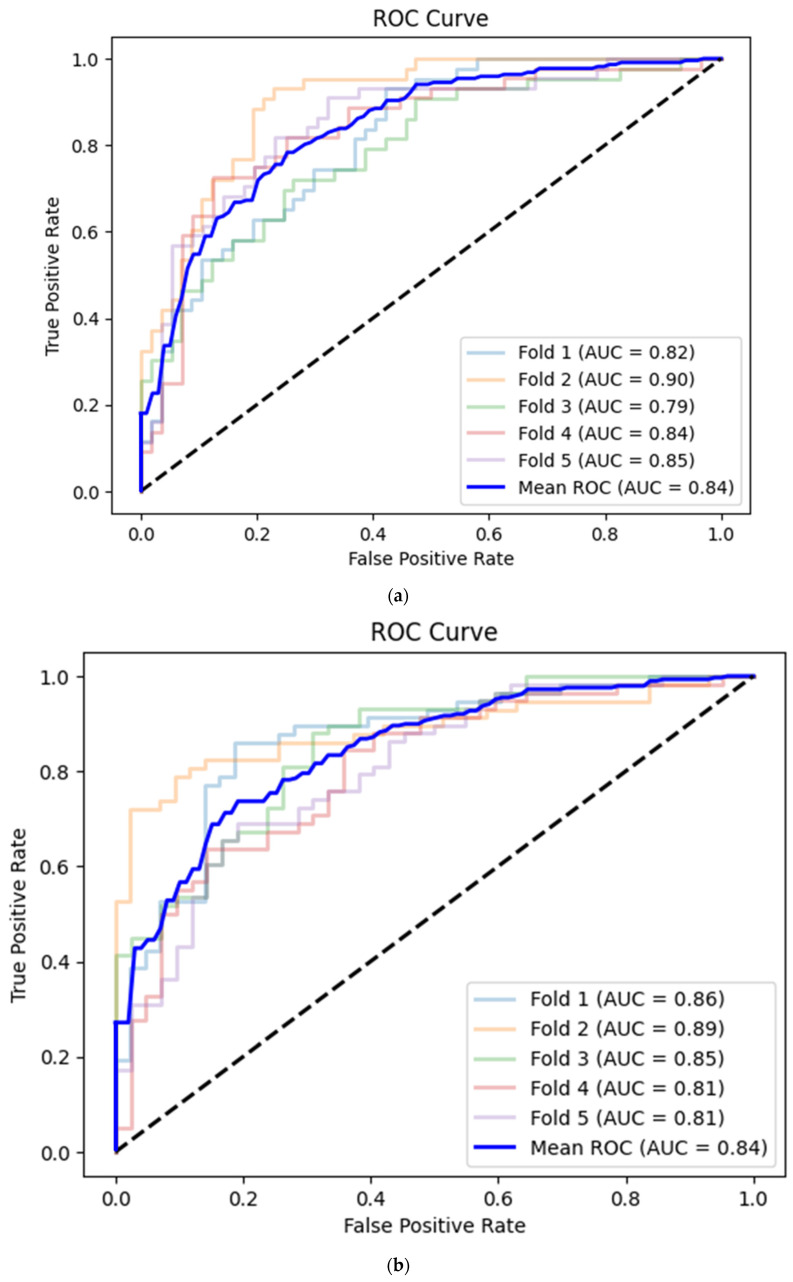

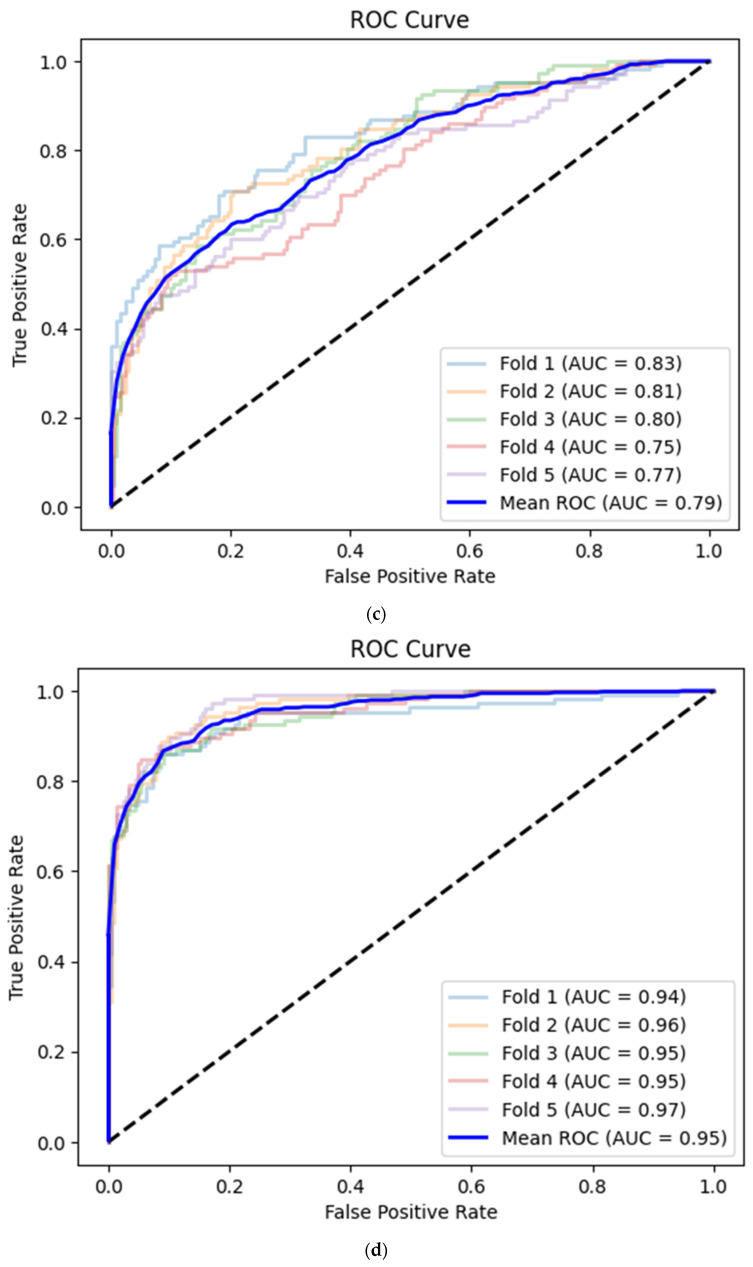
(**a**) Best convolutional neural network model for CAMUS apical 2-chamber view image quality classification with Receiver Operating Curve. AUC—area under the curve. (**b**) Best convolutional neural network model for CAMUS apical 4-chamber view image quality classification with Receiver Operating Curve. AUC—area under the curve. (**c**) Best convolutional neural network model for Unity Imaging parasternal long-axis view image quality classification with Receiver Operating Curve. AUC—area under the curve. (**d**) Best convolutional neural network model for Unity Imaging parasternal long-axis view image quality classification with Receiver Operating Curve. AUC—area under the curve.

A convolutional neural network based on the corresponding image dataset achieved on average 0.76 (95% CI 0.72–0.79) accuracy, 0.64 (95% CI 0.59–0.68) sensitivity, 0.85 (95% CI 0.82–0.88) specificity and 0.84 (95% CI 0.81–0.87) AUC (Table 3, Figure 5a). There were no statistically significant differences between the deep learning and machine learning models (*p* > 0.05 for all metrics, Table 4).

### 3.2. CAMUS Apical 4-Chamber View

The best average prediction accuracy for the histogram dataset calculated from the apical 4-chamber view was achieved by a Random Forest model with a histogram grid length of 10, this time with 0.74 (95% CI 0.70–0.78) accuracy, 0.81 (95% CI 0.77–0.84) sensitivity, 0.65 (95% CI 0.61–0.70) specificity and 0.81 (95% CI 0.77–0.84) AUC (Table 2, Figure 4b).

The prediction mean accuracy of the CNN for the apical 4-chamber view images was equal to 0.74 (95% CI 0.70–0.78) with 0.83 (95% CI 0.79–0.86) sensitivity, 0.63 (95% CI 0.59–0.67) specificity and 0.84 (95% CI 0.81–0.87) AUC (Table 3, Figure 5b).

There were no statistically significant differences between the deep learning and machine learning models (*p* > 0.05 for all evaluated metrics, Table 4).

### 3.3. Unity Imaging Parasternal Long-Axis View

For the best machine learning model, the average classification accuracy of image quality for the histogram dataset was equal to 0.83 (95% CI 0.81–0.84) with 0.61 (95% CI 0.58–0.63) sensitivity, 0.94 (95% CI 0.93–0.95) specificity and 0.88 (95% CI 0.86–0.89) AUC. This model was based on an XGBoost classifier with a histogram grid length of 10 (Table 2, Figure 4c).

A convolutional neural network based on the corresponding image dataset achieved on average 0.75 (95% CI 0.73–0.77) image quality classification accuracy, 0.55 (95% CI 0.52–0.57) sensitivity, 0.86 (95% CI 0.84–0.88) specificity and 0.79 (95% CI 0.77–0.81) AUC (Table 3, Figure 5c).

There were statistically significant differences between the models (*p* < 0.001 for all evaluated metrics; Table 4).

### 3.4. Unity Imaging Apical 4-Chamber View

Among machine learning models, the best prediction mean accuracy for the histogram dataset calculated from the apical 4-chamber view was also achieved by an XGBoost model with a histogram grid length of 10, this time with 0.92 (95% CI 0.90–0.93) accuracy, 0.82 (95% CI 0.80–0.84) sensitivity, 0.97 (95% CI 0.96–0.97) specificity and 0.96 (95% CI 0.95–0.97) AUC (Table 2, Figure 4d).

The average prediction accuracy of the CNN for the apical 4-chamber view images was equal to 0.89 (95% CI 0.87–0.90) with 0.79 (95% CI 0.77–0.81) sensitivity, 0.94 (95% CI 0.92–0.95) specificity and 0.95 (95% CI 0.94–0.96) AUC (Table 3, Figure 5d).

There were statistically significant differences between the models in terms of mean accuracy (0.92 for the machine learning model versus 0.89 for CNN, *p* = 0.008) and specificity (0.97 for the machine learning model versus 0.94 for CNN, *p* < 0.001; Table 4).

### 3.5. Trends in Predictions

With the use of the machine learning algorithms, the mean prediction time for one block of histograms derived from one image was below 1 ms.

For the convolutional neural network, the mean prediction time for one image was 304.1 ms with a standard deviation of 24.9 ms. For a batch of ten images, the mean prediction time per image was 162.6 ms with a standard deviation of 18.2 ms.

Of note, algorithms like AdaBoost and the Support Vector classifier also had very good performance. Usually, their classification accuracy is only about 1–4% lower than the prediction accuracy of the best model (Supplementary Material).

Interestingly, the developed algorithms had very high specificity values for the histograms derived from the Unity Imaging dataset (over 90%). Moreover, for the majority of the tested cases, with the lengthening of the histogram grid (from 1 to 10), all performance metrics gradually increased. The largest performance boost was usually for the transition between grid lengths of 1 and 3. However, no one common grid length would define a plateau after which the increase would stabilize (Supplementary Material).

The timing of the predictions was performed using Google Colab software (Python 3.10) and a Tesla T4 graphical processing unit (16 GB Video RAM).

### 3.6. Open-Source Availability of the Best Models

The best models that support the findings of this study are available from the corresponding author, Wojciech Nazar, upon reasonable request. They can be used for scientific purposes free of charge.

## 4. Discussion

This study aimed to develop, validate, and compare machine learning and deep learning algorithms for accurate, automated and efficient assessment of transthoracic echocardiogram image quality on two open-source datasets.

Classic machine learning models were trained on blocks of histograms calculated from the original raw image. Deep learning algorithms analyzed raw images based on a convolutional neural network using the pre-trained EfficientNetB4 architecture.

In the presented classification problem, classic machine learning can achieve prediction accuracy comparable to or greater than the “gold standard” convolutional neural networks in image data classification.

### 4.1. Accuracy of Predictions

Both classic machine learning models and convolutional neural networks predicted the image quality of a transthoracic echocardiogram with at least 0.74 accuracy, reaching over 0.90 for some datasets (Table 4). The echocardiogram image quality, the end-point for the prediction models, was assessed differently in the CAMUS and Unity Imaging datasets (Figure 1). Images in CAMUS were directly labelled as “good”, “medium” or “poor” by an experienced cardiologist. On the contrary, every image in the Unity Imaging dataset initially contains a set of labels described as “off”, “blurred”, or “point/curve” for clinically relevant structures. Next, for this study, sets of these labels were changed into scores to describe images of “good” or “poor” quality. Even though two very different methods were used for image quality assessment, the best algorithms could predict image quality with an accuracy from 0.74 to over 0.90 (Table 3). Therefore, classical machine learning and deep learning are valuable methodologies for image quality assessment (Figure 2, Figure 3).

Interestingly, there were discrepancies between the best accuracy values of machine learning and deep learning for the Unity Imaging dataset. Images in the parasternal long-axis view were predicted with 0.83 (95% CI 0.81–0.84) accuracy using the XGBoost classifier and 0.75 (95% CI 0.73–0.77) accuracy with the use of a convolutional neural network (*p* < 0.001, Table 4). The image quality was predicted for the apical 4-chamber dataset with 0.92 (95% CI 0.90–0.93) accuracy using the XGBoost model and 0.89 (95% CI 0.87–0.90) accuracy with the use of a convolutional neural network (*p* = 0.008). For the CAMUS dataset, the differences between the machine learning and deep learning models were lower. For apical 2-chamber views, the mean accuracy of the Random Forest classifier and convolutional neural network were the same (0.76; 95% CI 0.72–0.79; *p* = 1.000, Table 3). Further on, for the CAMUS apical 4-chamber dataset, the image quality was also predicted with the same accuracy for both machine learning and deep learning techniques (0.74; 95% CI 0.70–0.78; *p* = 1.000).

Overall, when tested on the Unity Imaging dataset, the machine learning model trained on block histograms calculated from raw images outperformed gold-standard convolutional neural networks trained on raw images. When tested on images from the CAMUS dataset, machine learning and deep learning models achieved equal performance. Nevertheless, both methods seem to be useful, and it is advised to train both classic machine learning and deep learning algorithms on any available dataset. This will allow for robust and reliable model development, validation, and comparison. Eventually, a model with the best accuracy and/or clinical applicability will be selected.

### 4.2. Block Histograms: A Novel Approach for Image Quality Analysis

This study is a proof of concept of a novel approach to training classic machine learning algorithms on block histograms calculated from raw images. The advantages of this approach include easier and faster training of the model and much shorter prediction times of below 1 ms per image. The high accuracy of this method can be explained by the fact that histograms are calculated from an image in its native resolution. Moreover, the aim of the algorithm is not to detect exact shapes (which is one of the principles of computer vision based on convolutional neural networks) but to find general relationships between the proportions of black and white pixels. These relationships can probably be more easily modelled with the use of block histograms, which (1) transform raw image pixel data into tabular data and (2) are the preprocessing step, which extracts relevant information (proportions of different brightness values) for the final algorithm. Overall, it contributes to the high prediction accuracy of the new approach.

### 4.3. Factors Determining the Accuracy of the Models

However, it must be remembered that a model’s prediction accuracy depends mainly on three factors: the quality of the input data, mathematical modelling and, most importantly, the quality of the end-point assessment method. The image quality assessment method applied for the Unity Imaging (sum of scores based on the visibility of clinically relevant anatomical structures) is more methodologically objective than the direct and ad hoc image quality labelling in the CAMUS dataset. Thus, the differences in the accuracy of predictions may not only result from the mathematical principles on which various machine and deep learning algorithms are based but also from the less accurate labelling of the endpoint. If the end-point assessment method is less objective, there might be more noise between the input data and the predicted label. Thus, it might be more challenging for the algorithm to learn mathematical relationships between the input data (images or their histograms) and various classes of the forecasted endpoint (good or poor image quality). In addition to that, the sample size in CAMUS datasets was lower (n = 500/500) than in the Unity Imaging dataset (n = 1559/1531), which may also partially explain the lower accuracy of the models trained on data from the CAMUS repository (Figure 1, Table 1).

### 4.4. Sensitivity and Specificity of the Algorithms

Sensitivity was defined as the ability of the algorithm to correctly detect images of good quality [28]. Specificity was defined as the ability of the model to correctly classify images of poor quality correctly [28]. Except for the CAMUS apical 4-chamber images, in all other datasets, the proportion of images of poor quality was larger. A consequence of this trend is also visible in the slightly skewed sensitivity and specificity of the predictions. The sensitivity of the CAMUS apical 4-chamber images was over 80% for both machine learning and deep learning models. However, the specificity was lower and equal to 63–65% for the best models. Therefore, the model sometimes identified poor-quality images as good-quality images which resulted in a higher false positive rate. In contrast, for the rest of the datasets, both approaches resulted in prediction models with over 80% specificity in most cases. However, for the CAMUS apical 2-chamber and Unity Imaging parasternal long-axis datasets the sensitivity was relatively low (64–68% and 0.55–0.61%, respectively). Therefore, these models tended to identify good-quality images very reliably (high specificity of over 80%), but sometimes, good-quality images were falsely predicted as poor-quality images, which resulted in relatively low sensitivity and a higher false-negative rate.

For the developed models, the values of AUC were usually slightly higher than the reported corresponding accuracies (Table 4). Thus, the models seem to provide rather balanced prediction capabilities from the statistical point of view [27,29].

Since the automated LVEF and GLS calculation principles are based on the semi-automatic detection of endocardial borders, which implements computer vision methods, LVEF/GLS measurement can be biased and have higher variability due to poor image quality [1,2,27]. Thus, high specificity will be helpful in clinical practice, as it will reliably detect images of poor quality that should be used with caution for advanced AI-guided measurements.

### 4.5. Comparison with Other Studies

Labs et al. predicted transthoracic echocardiogram image quality with 96.2% accuracy using a dataset of 33,784 images in parasternal long-axis and apical 4-chamber views [19]. Loung et al. achieved 87.0% in image quality prediction using a dataset of 14,086 images in nine views [18]. Either developed algorithms based on convolutional neural networks are considered the gold standard of computer vision [20,30]. Our study analyzed parasternal long-axis, apical 4-chamber and apical 2-chamber views (sample size from 500 to 1559 images, Figure 1, Table 1). Classic machine learning algorithms predicted image quality with 0.74 to 0.92 accuracy (AUC 0.81 to 0.96), whereas convolutional neural networks achieved between 0.74 and 0.89 prediction accuracy (AUC 0.79 to 0.95; Table 4). Thus, the accuracy on some datasets was lower than the one presented by Labs et al. and comparable to the one described by Loung et al. However, our study was based on datasets with a much smaller sample size, which can explain the differences in the compared accuracies.

## 5. Limitations

In our study, two open-source echocardiogram image datasets were compared. The datasets could not be fused because they initially utilized a different methodology for image quality assessment. Thus, it was not possible.to develop one machine and/or deep learning algorithm to fit all available data.

Moreover, both datasets provided few clinically relevant open-source individual patient data about the patients from which the echocardiogram images came [15,26]. This is not only the case with these particular dataset sources but also other studies and open-source data on which machine learning studies are based [31,32]. Moreover, even studies that use their private data sometimes provide scarce clinical characteristics regarding the inclusion/exclusion criteria of the patients included in the study [19,32]. They usually focus on advanced mathematical modelling and the performance of AI algorithms. However, it is not only the performance but also the clinical applicability of the algorithm that makes it valuable for use in daily clinical practice [33]. Thus, future studies should focus on reporting not only open-source high-quality image datasets but also individual patient data so that the studies based on these open-source data would analyze the mathematical principles and accuracy of the models and the clinical characteristics of the studied population. Moreover, the open-source availability of echocardiogram image classification algorithms developed in other studies is also limited [17,18,19]. Thus, performing a head-to-head validation and comparison of the newly developed model with the existing ones was not possible.

Further, the heterogeneous nature of image quality definition is also a limitation in our study. The variability in how image quality is defined and assessed across the analyzed datasets could impact the performance of our model. Image quality is subjectively evaluated, with different observers potentially having varied perceptions of what constitutes a “high-quality” image. Therefore, to address this issue, future projects should focus on developing more standardized and universally accepted definitions and assessment methods for image quality and incorporating a diverse range of images from various sources and conditions into training datasets to improve the robustness and generalizability of echocardiogram image quality assessment models.

Another limitation of this study is the lack of comparison between the algorithm’s performance and inter-observer variability. Understanding how the algorithms’ accuracy and uncertainty compare to the variability between different human observers would provide an important context for its reliability. However, such data were not available in the original datasets. Future research should aim to include this comparison to offer a more comprehensive evaluation of the algorithm’s performance.

Ultimately, numerous algorithms, particularly advanced deep learning models (convolutional neural networks), are characterized as “black box algorithms,” and the transparency of their predictive mechanisms remains unknown. Even the less advanced machine learning models only partially explain the most predictive features. Given the opacity of how the model arrives at its output, there is ongoing discussion about whether such algorithms should be integrated into routine clinical practice [33,34].

## 6. Conclusions

Both classic machine learning models based on image histograms and convolutional neural networks trained on raw images are accurate and valuable automated echocardiogram image quality assessment methods. However, the training of machine learning algorithms is easier and faster, and the prediction time per image is shorter in comparison to convolutional neural networks. In addition, this study is a proof of concept of a novel and accurate method of training classic machine learning algorithms on block histograms calculated from raw images. Automated echocardiogram image quality assessment methods may provide additional relevant information to the echocardiographer in daily clinical practice and may improve reliability in clinical decision making.

## Figures and Tables

**Table 1:** Characteristics of CAMUS and Unity Imaging datasets.

Dataset (View)	Characteristics	Mean	Median	Min	Max	Standard Deviation
CAMUS (apical 2-chamber)	image height in pixels	984.0	973.0	584.0	1945.0	157.8
	image width in pixels	600.9	591.0	323.0	1181.0	104.5
	mean brightness	50.2	49.3	14.8	104.0	12.2
	number of pixels	606,740.2	575,043.0	206,736.0	229,7045.0	202,752.9
	width-to-height ratio	0.6	0.6	0.5	0.9	0.0
CAMUS (apical 4-chamber)	image height in pixels	985.2	973.0	584.0	1945.0	160.8
	image width in pixels	599.4	591.0	323.0	1181.0	105.6
	mean brightness	50.5	49.8	20.0	95.0	11.8
	number of pixels	606,721.2	575,043.0	206,736.0	229,7045.0	205,842.2
	width-to-height ratio	0.6	0.6	0.5	0.9	0.0
Unity Imaging (parasternal long-axis)	image height in pixels	554.7	600.0	300.0	768.0	81.1
	image width in pixels	749.3	800.0	400.0	1024.0	95.7
	mean brightness	17.6	17.0	3.7	42.1	6.5
	number of pixels	423,200.0	480,000.0	120,000.0	786,432.0	110,102.8
	width-to-height ratio	1.4	1.3	1.3	1.5	0.1
Unity Imaging (apical 4-chamber)	image height in pixels	548.0	600.0	300.0	768.0	101.2
	image width in pixels	754.4	800.0	400.0	1024.0	108.9
	mean brightness	18.7	17.4	5.2	71.4	7.7
	number of pixels	424,257.0	480,000.0	120,000.0	786,432.0	140,175.5
	width-to-height ratio	1.4	1.3	1.3	1.5	0.1

**Table 2:** Machine learning models with the best classification performance. The average and standard deviation are derived from 5-fold cross-validation. AUC—area under the curve.

Dataset—Best Model (Grid Length)	Evaluation Metrics	Fold during 5-Fold Cross-Validation					Average	Standard Deviation
		1	2	3	4	5		
CAMUS apical 2-chamber view—Random Forest classifier (grid length 5)	Accuracy	0.72	0.79	0.79	0.71	0.78	0.76	0.04
	Sensitivity	0.68	0.70	0.70	0.63	0.70	0.68	0.03
	Specificity	0.75	0.86	0.86	0.77	0.84	0.82	0.05
	AUC	0.80	0.86	0.83	0.78	0.83	0.82	0.03
	True negative	42	48	49	44	48	46.20	3.03
	False positive	14	8	8	13	9	10.40	2.88
	False negative	14	13	13	16	13	13.80	1.30
	True positive	30	31	30	27	30	29.60	1.52
CAMUS apical 4-chamber view—Random Forest classifier (grid length 10)	Accuracy	0.71	0.80	0.71	0.78	0.72	0.74	0.04
	Sensitivity	0.70	0.82	0.81	0.86	0.84	0.81	0.06
	Specificity	0.72	0.77	0.57	0.67	0.55	0.65	0.09
	AUC	0.77	0.86	0.78	0.83	0.78	0.81	0.04
	True negative	31	33	24	28	23	27.80	4.32
	False positive	12	10	18	14	19	14.60	3.85
	False negative	17	10	11	8	9	11.00	3.54
	True positive	40	47	47	50	49	46.60	3.91
Unity Imaging parasternal long-axis view—XGBoost classifier (grid length 10)	Accuracy	0.80	0.81	0.84	0.85	0.83	0.83	0.02
	Sensitivity	0.53	0.57	0.63	0.67	0.64	0.61	0.06
	Specificity	0.94	0.94	0.95	0.95	0.93	0.94	0.01
	AUC	0.85	0.86	0.89	0.91	0.86	0.88	0.03
	True negative	189	189	190	189	185	188.40	1.95
	False positive	12	12	10	11	15	12.00	1.87
	False negative	50	45	39	35	38	41.40	6.02
	True positive	56	60	67	71	68	64.40	6.19
Unity Imaging apical 4-chamber view—XGBoost classifier (grid length 8)	Accuracy	0.92	0.93	0.92	0.90	0.91	0.92	0.01
	Sensitivity	0.79	0.88	0.80	0.80	0.82	0.82	0.03
	Specificity	0.98	0.96	0.99	0.95	0.96	0.97	0.02
	AUC	0.96	0.98	0.97	0.95	0.96	0.96	0.01
	True negative	201	196	203	196	197	198.60	3.21
	False positive	4	9	3	10	8	6.80	3.11
	False negative	22	13	21	21	19	19.20	3.63
	True positive	85	94	85	85	87	87.20	3.90

**Table 3:** Convolutional neural networks with the best classification performance. The average and standard deviation are derived from 5-fold cross-validation. AUC—area under the curve.

Dataset	Evaluation Metrics	Fold during 5-Fold Cross-Validation					Average	Standard Deviation
		1	2	3	4	5		
CAMUS apical 2-chamber view	Accuracy	0.72	0.80	0.72	0.79	0.76	0.76	0.04
	Sensitivity	0.49	0.77	0.58	0.64	0.70	0.64	0.11
	Specificity	0.89	0.82	0.82	0.91	0.80	0.85	0.05
	AUC	0.82	0.90	0.79	0.84	0.85	0.84	0.04
	True negative	51	47	47	51	45	48.20	2.68
	False positive	6	10	10	5	11	8.40	2.70
	False negative	22	10	18	16	13	15.80	4.60
	True positive	21	33	25	28	31	27.60	4.77
CAMUS apical 4-chamber view	Accuracy	0.82	0.69	0.74	0.76	0.71	0.74	0.05
	Sensitivity	0.89	0.95	0.74	0.84	0.71	0.83	0.10
	Specificity	0.72	0.35	0.74	0.64	0.71	0.63	0.16
	AUC	0.86	0.89	0.85	0.81	0.81	0.84	0.04
	True negative	31	15	31	27	30	26.80	6.80
	False positive	12	28	11	15	12	15.60	7.09
	False negative	6	3	15	9	17	10.00	5.92
	True positive	51	54	43	49	41	47.60	5.46
Unity Imaging parasternal long-axis view	Accuracy	0.78	0.75	0.76	0.75	0.72	0.75	0.02
	Sensitivity	0.71	0.35	0.55	0.53	0.60	0.55	0.13
	Specificity	0.81	0.97	0.88	0.88	0.78	0.86	0.07
	AUC	0.83	0.81	0.80	0.75	0.77	0.79	0.03
	True negative	163	194	175	175	156	172.60	14.47
	False positive	38	6	25	25	45	27.80	14.92
	False negative	31	69	48	50	42	48.00	13.87
	True positive	75	37	58	56	63	57.80	13.77
Unity Imaging apical 4-chamber view	Accuracy	0.88	0.88	0.89	0.89	0.89	0.89	0.01
	Sensitivity	0.79	0.68	0.78	0.86	0.84	0.79	0.07
	Specificity	0.93	0.98	0.95	0.91	0.92	0.94	0.03
	AUC	0.94	0.96	0.95	0.95	0.97	0.95	0.01
	True negative	190	201	195	188	189	192.60	5.41
	False positive	15	4	11	18	16	12.80	5.54
	False negative	22	34	23	15	17	22.20	7.40
	True positive	85	73	83	91	89	84.20	7.01

**Table 4:** Statistical analysis of the best models. The average values of evaluation metrics are presented. CNN—convolutional neural network. AUC—area under the curve. 95% CI—95% confidence interval.

Dataset and Best Models	Evaluation Metrics	CNN (Deep Learning)	Machine Learning	*p*-Value
CAMUS apical 2-chamber view: CNN versus Random Forest classifier (grid length 5)	Accuracy	0.76 (95% CI 0.72–0.79)	0.76 (95% CI 0.72–0.79)	1.000
	Sensitivity	0.64 (95% CI 0.59–0.68)	0.68 (95% CI 0.64–0.72)	0.140
	Specificity	0.85 (95% CI 0.82–0.88)	0.82 (95% CI 0.78–0.85)	0.155
	AUC	0.84 (95% CI 0.81–0.87)	0.82 (95% CI 0.78–0.85)	0.406
CAMUS apical 4-chamber view: CNN versus Random Forest classifier (grid length 10)	Accuracy	0.74 (95% CI 0.70–0.78)	0.74 (95% CI 0.70–0.78)	1.000
	Sensitivity	0.83 (95% CI 0.79–0.86)	0.81 (95% CI 0.77–0.84)	0.503
	Specificity	0.63 (95% CI 0.59–0.67)	0.65 (95% CI 0.61–0.70)	0.513
	AUC	0.84 (95% CI 0.81–0.87)	0.81 (95% CI 0.77–0.84)	0.138
Unity Imaging parasternal long-axis view: CNN versus XGBoost classifier (grid length 10)	Accuracy	0.75 (95% CI 0.73–0.77)	0.83 (95% CI 0.81–0.84)	<0.001
	Sensitivity	0.55 (95% CI 0.52–0.57)	0.61 (95% CI 0.58–0.63)	<0.001
	Specificity	0.86 (95% CI 0.84–0.88)	0.94 (95% CI 0.93–0.95)	<0.001
	AUC	0.79 (95% CI 0.77–0.81)	0.88 (95% CI 0.86–0.89)	<0.001
Unity Imaging apical 4-chamber view: CNN versus XGBoost classifier (grid length 8)	Accuracy	0.89 (95% CI 0.87–0.90)	0.92 (95% CI 0.90–0.93)	0.008
	Sensitivity	0.79 (95% CI 0.77–0.81)	0.82 (95% CI 0.80–0.84)	0.054
	Specificity	0.94 (95% CI 0.92–0.95)	0.97 (95% CI 0.96–0.97)	<0.001
	AUC	0.95 (95% CI 0.94–0.96)	0.96 (95% CI 0.95–0.97)	0.172

## Data Availability

Data and algorithms that support the findings of this study are available from the corresponding author, WN, upon reasonable request.

## Supplementary Materials

The following supporting information can be downloaded at: https://www.mdpi.com/article/10.3390/life14060761/s1, Table S1: Detailed performance results of the developed machine learning models.

## References

[1] Nagata Y., Kado Y., Onoue T., Otani K., Nakazono A., Otsuji Y., Takeuchi M. (2018). Impact of Image Quality on Reliability of the Measurements of Left Ventricular Systolic Function and Global Longitudinal Strain in 2D Echocardiography. Echo Res. Pract..

[2] Huang K.C., Huang C.S., Su M.Y., Hung C.L., Ethan Tu Y.C., Lin L.C., Hwang J.J. (2021). Artificial Intelligence Aids Cardiac Image Quality Assessment for Improving Precision in Strain Measurements. JACC Cardiovasc. Imaging.

[3] Sengupta P.P., Marwick T.H. (2021). Enforcing Quality in Strain Imaging Through AI-Powered Surveillance. JACC Cardiovasc. Imaging.

[4] Al Saikhan L., Park C., Hughes A.D. (2019). Reproducibility of Left Ventricular Dyssynchrony Indices by Three-Dimensional Speckle-Tracking Echocardiography: The Impact of Sub-Optimal Image Quality. Front. Cardiovasc. Med..

[5] Johnson K.W., Torres Soto J., Glicksberg B.S., Shameer K., Miotto R., Ali M., Ashley E., Dudley J.T. (2018). Artificial Intelligence in Cardiology. J. Am. Coll. Cardiol..

[6] Bizopoulos P., Koutsouris D. (2019). Deep Learning in Cardiology. IEEE Rev. Biomed. Eng..

[7] He B., Kwan A.C., Cho J.H., Yuan N., Pollick C., Shiota T., Ebinger J., Bello N.A., Wei J., Josan K. (2023). Blinded, Randomized Trial of Sonographer versus AI Cardiac Function Assessment. Nature.

[8] Harjoko R.P., Sobirin M.A., Uddin I., Bahrudin U., Maharani N., Herminingsih S., Tsutsui H. (2022). Trimetazidine Improves Left Ventricular Global Longitudinal Strain Value in Patients with Heart Failure with Reduced Ejection Fraction Due to Ischemic Heart Disease. Drug Discov. Ther..

[9] Mazzetti S., Scifo C., Abete R., Margonato D., Chioffi M., Rossi J., Pisani M., Passafaro G., Grillo M., Poggio D. (2020). Short-Term Echocardiographic Evaluation by Global Longitudinal Strain in Patients with Heart Failure Treated with Sacubitril/Valsartan. ESC Heart Fail..

[10] Sławiński G., Hawryszko M., Liżewska-Springer A., Nabiałek-Trojanowska I., Lewicka E. (2023). Global Longitudinal Strain in Cardio-Oncology: A Review. Cancers.

[11] Ramesh A.N., Kambhampati C., Monson J.R.T., Drew P.J. (2004). Artificial Intelligence in Medicine. Ann. R. Coll. Surg. Engl..

[12] Galmarini C.M., Lucius M. (2020). Artificial Intelligence: A Disruptive Tool for a Smarter Medicine. Eur. Rev. Med. Pharmacol. Sci..

[13] Wagner M.W., Namdar K., Biswas A., Monah S., Khalvati F., Ertl-Wagner B.B. (2021). Radiomics, Machine Learning, and Artificial Intelligence—What the Neuroradiologist Needs to Know. Neuroradiology.

[14] Topol E.J. (2019). High-Performance Medicine: The Convergence of Human and Artificial Intelligence. Nat. Med..

[15] Howard J.P., Stowell C.C., Cole G.D., Ananthan K., Demetrescu C.D., Pearce K., Rajani R., Sehmi J., Vimalesvaran K., Kanaganayagam G.S. (2021). Automated Left Ventricular Dimension Assessment Using Artificial Intelligence Developed and Validated by a UK-Wide Collaborative. Circ. Cardiovasc. Imaging.

[16] Chao P.K., Wang C.L., Chan H.L. (2012). An Intelligent Classifier for Prognosis of Cardiac Resynchronization Therapy Based on Speckle-Tracking Echocardiograms. Artif. Intell. Med..

[17] Dong J., Liu S., Liao Y., Wen H., Lei B., Li S., Wang T. (2020). A Generic Quality Control Framework for Fetal Ultrasound Cardiac Four-Chamber Planes. IEEE J. Biomed. Health Inform..

[18] Luong C., Liao Z., Abdi A., Girgis H., Rohling R., Gin K., Jue J., Yeung D., Szefer E., Thompson D. (2021). Automated Estimation of Echocardiogram Image Quality in Hospitalized Patients. Int. J. Cardiovasc. Imaging.

[19] Labs R.B., Vrettos A., Loo J., Zolgharni M. (2023). Automated Assessment of Transthoracic Echocardiogram Image Quality Using Deep Neural Networks. Intell. Med..

[20] Krizhevsky A., Sutskever I., Hinton G.E. ImageNet Classification with Deep Convolutional Neural Networks. Proceedings of the Advances in Neural Information Processing Systems 25 (NIPS 2012).

[21] Tandon A., Mohan N., Jensen C., Burkhardt B.E.U., Gooty V., Castellanos D.A., McKenzie P.L., Zahr R.A., Bhattaru A., Abdulkarim M. (2021). Retraining Convolutional Neural Networks for Specialized Cardiovascular Imaging Tasks: Lessons from Tetralogy of Fallot. Pediatr. Cardiol..

[22] Tan M., Le Q.V. EfficientNet: Rethinking Model Scaling for Convolutional Neural Networks. Proceedings of the 36th International Conference on Machine Learning.

[23] Cikes M., Sanchez-Martinez S., Claggett B., Duchateau N., Piella G., Butakoff C., Pouleur A.C., Knappe D., Biering-Sørensen T., Kutyifa V. (2019). Machine Learning-Based Phenogrouping in Heart Failure to Identify Responders to Cardiac Resynchronization Therapy. Eur. J. Heart Fail..

[24] Howell S.J., Stivland T., Stein K., Ellenbogen K.A., Tereshchenko L.G. (2021). Using Machine-Learning for Prediction of the Response to Cardiac Resynchronization Therapy: The SMART-AV Study. JACC Clin. Electrophysiol..

[25] Pietka E. (2000). Image Standardization in PACS. Handbook of Medical Imaging.

[26] Leclerc S., Smistad E., Pedrosa J., Ostvik A., Cervenansky F., Espinosa F., Espeland T., Berg E.A.R., Jodoin P.M., Grenier T. (2019). Deep Learning for Segmentation Using an Open Large-Scale Dataset in 2D Echocardiography. IEEE Trans. Med. Imaging.

[27] Sengupta P.P., Shrestha S., Berthon B., Messas E., Donal E., Tison G.H., Min J.K., D’hooge J., Voigt J.U., Dudley J. (2020). Proposed Requirements for Cardiovascular Imaging-Related Machine Learning Evaluation (PRIME): A Checklist: Reviewed by the American College of Cardiology Healthcare Innovation Council. JACC Cardiovasc. Imaging.

[28] Monaghan T.F., Rahman S.N., Agudelo C.W., Wein A.J., Lazar J.M., Everaert K., Dmochowski R.R. (2021). Foundational Statistical Principles in Medical Research: Sensitivity, Specificity, Positive Predictive Value, and Negative Predictive Value. Medicina.

[29] Sajjadian M., Lam R.W., Milev R., Rotzinger S., Frey B.N., Soares C.N., Parikh S.V., Foster J.A., Turecki G., Müller D.J. (2021). Machine Learning in the Prediction of Depression Treatment Outcomes: A Systematic Review and Meta-Analysis. Psychol. Med..

[30] Jones D.S., Podolsky S.H. (2015). The History and Fate of the Gold Standard. Lancet.

[31] Puyol-Antón E., Sidhu B.S., Gould J., Porter B., Elliott M.K., Mehta V., Rinaldi C.A., King A.P. (2022). A Multimodal Deep Learning Model for Cardiac Resynchronisation Therapy Response Prediction. Med. Image Anal..

[32] Degerli A., Kiranyaz S., Hamid T., Mazhar R., Gabbouj M. (2024). Early Myocardial Infarction Detection over Multi-View Echocardiography. Biomed. Signal Process Control.

[33] Nazar W., Szymanowicz S., Nazar K., Kaufmann D., Wabich E., Braun-Dullaeus R., Daniłowicz-Szymanowicz L. (2023). Artificial Intelligence Models in Prediction of Response to Cardiac Resynchronization Therapy: A Systematic Review. Heart Fail. Rev..

[34] Poon A.I.F., Sung J.J.Y. (2021). Opening the Black Box of AI-Medicine. J. Gastroenterol. Hepatol..

